# Axial Compression of BFRP Spiral Strip–PVC Tube Confined Fiber-Recycled Concrete: Experiment and FEM Analysis

**DOI:** 10.3390/ma18153431

**Published:** 2025-07-22

**Authors:** Jiaxing Tian, Huaxin Liu, Genjin Liu, Wenyu Wang, Jiuwen Bao

**Affiliations:** 1 College of Civil and Architectural Engineering, Liaoning University of Technology, Jinzhou 121001, China; 13190439340@163.com (J.T.); lgliuhuaxin@163.com (H.L.); wwenyu528@163.com (W.W.); 2 School of Civil Engineering, NingboTech University, Ningbo 315100, China; 3 School of Civil Engineering, Qingdao University of Technology, Qingdao 266520, China; baojiuwen@qut.edu.cn

**Keywords:** spiral strip, recycled aggregate concrete, FEM, composite structure, strength model

## Abstract

The use of short cylinders of recycled aggregate concrete (RAC) reinforced with basalt fiber-reinforced polymer (BFRP) circumferential strips and polyvinyl chloride (PVC) tubes has been proven effective in previous studies. However, BFRP circumferential strips are cumbersome to install and do not ensure the integrity of the BFRP strips. Therefore, this study investigates axial compression experiments on RAC short cylinders reinforced with BFRP spiral strips and PVC tubes. A combination of experimental studies, finite element simulations, and theoretical analyses revealed that the winding angle and spacing of BFRP strips significantly affect the load-bearing capacity and ductility of the restrained specimens. Additionally, an improved strength model was developed based on an existing model. When evaluated using both computational and experimental results, the equations generated in this study showed an average error of less than 10%. The findings indicate that the composite structure provides effective reinforcement and offers valuable reference information for practical applications.

## 1. Introduction

The disposal of construction waste poses significant challenges worldwide due to its environmental impact and growing demand for landfill sites [1]. Recycled Aggregate Concrete (RAC), a material derived from building waste (e.g., waste concrete, bricks), has attracted increasing attention and application in recent years [2,3]. RAC can partially replace natural sand and gravel and is widely used because of its high aggregate content (approximately 65–85% of its volume) [4]. Although the crushing and processing of waste materials to produce recycled aggregates (RAs) consume energy, several life cycle assessment (LCA) studies [5] show that the overall environmental impact of recycled aggregate concrete (RAC) is lower than that of conventional concrete. Key factors include shorter transport distances (0–40 km for RAs vs. 0.3–280 km for natural aggregates) and carbon sequestration through carbonation. However, the environmental benefits of RAC may decrease beyond a “critical distance” due to transportation impacts. Overall, RAC helps reduce the demand for virgin aggregates and avoids landfill disposal, contributing significantly to its sustainability. This is due to the reduced demand for natural aggregates and the diversion of construction waste from landfills, both of which contribute to improved sustainability. However, the performance of RAC is inherently limited, with lower strength, reduced ductility, and poorer durability compared to conventional concrete, as it is made from construction waste [6,7].

To address these issues, Das et al. [8] proposed that blending polypropylene fibers into RAC can effectively improve its splitting and flexural strengths. Htet et al. [9] suggested that the durability of RAC can be enhanced by using mixed fibers. Wang et al. [10] demonstrated that incorporating fiber reinforcement without altering the recycled aggregate can enhance the performance of RAC. On the other hand, some researchers have used external constraints, such as FRP-PVC composite structures, to reinforce RAC. These composite structures can improve the ductility, strength, and corrosion resistance of RAC [11,12]. This approach leverages the lightweight, high-strength, and easy-to-construct properties of FRP materials [13,14,15], along with the superior mechanical properties and corrosion resistance of PVC materials [12,16].

FRP-restrained concrete typically comes in two major forms: partially restrained and completely restrained [17]. Compared to fully restrained systems, partial FRP restraint offers advantages such as easier construction, lower material usage, and reduced cost [18,19,20]. Although partial FRP restraints are generally less effective than full restraints, several studies have shown that they can still provide adequate load-bearing capacity and deformation resistance [21,22]. There has been extensive research on partial FRP constraints. Liao et al. [23] studied concrete cylinders restrained by FRP helical strips and found that increasing the width and thickness of the FRP strips, as well as decreasing the helix angle, can enhance the ultimate axial stress–strain behavior of concrete. Ismail et al. [24] compared the performance of partially CFRP-restrained concrete cylinders with fully restrained and unconfined specimens, concluding that partial CFRP restraints with horizontal strips are sufficient. Furthermore, numerous researchers have explored FRP-PVC composite structures. Li et al. [25] observed that the FRP-PVC system significantly improved the load-bearing capacity and deformation resistance of rubberized concrete cylinders, with the peak axial stress–strain increasing as the spacing between the FRP strips decreased. Yu et al. [26,27] conducted experiments and finite element analyses on CFRP-PVC tube-restrained concrete cylinders, and their findings revealed that the PVC tube enables uniform stress transfer to the CFRP, resulting in high load-bearing capacity and excellent seismic performance.

Despite these advancements, research on the reinforcement of PVC tubes with BFRP strips remains limited, with most studies focusing on the annular restraint of BFRP strips. In addition to increasing the ultimate bearing capacity, Chu et al. [28] found that BFRP strip–PVC tube composite reinforcement improved the damage pattern of short fiber-recycled concrete cylinders. Wang et al. [29] analyzed the effectiveness of the BFRP-PVC composite reinforcement system and proposed a new equivalent model. In contrast, spiral strip reinforcement offers an alternative that ensures strip integrity, is more convenient, and maintains acceptable performance.

In this study, the mechanical properties of BFRP spiral strip–PVC tube confined RAC short cylinders under axial compression were investigated experimentally. The effects of BFRP strip winding angle and spacing on key mechanical properties, including load-bearing capacity, stress–strain behavior, and ductility, were analyzed. Additionally, ABAQUS numerical simulations were performed to validate the experimental findings. Based on the experimental results and existing strength models, a design model is proposed to predict the strength of BFRP spiral strip–PVC tube confined RAC short cylinders.

## 2. Experimental Programs

### 2.1. Design and Fabrication of Specimens

A total of 21 standard cylindrical specimens, each with a diameter of 150 mm and a height of 300 mm, were designed and fabricated in seven groups of three. Twelve of these specimens were BFRP spiral strip–PVC tube confined recycled aggregate concrete specimens, six were BFRP annular strip–PVC tube confined recycled aggregate concrete specimens, and three unconfined specimens served as controls. Information about the specimens is provided in Table 1. To prevent localized damage at the ends of the confined cylinders, the ends of the strip-confined specimens were additionally reinforced with BFRP circumferential strips, each with a bandwidth of 40 mm. The specimens are named according to specific rules: The number on the left indicates the recycled aggregate substitution rate, the number on the right represents the helix pitch, and the middle value denotes the winding angle.

In general, increasing the recycled aggregate replacement ratio (e.g., from 50% to 55% or 60%) may reduce concrete stiffness due to the lower strength and bonding quality of recycled aggregates. Conversely, lowering the ratio (e.g., to 40% or 30%) can enhance stiffness but decrease the use of sustainable materials [28,29]. Therefore, a 50% replacement rate was adopted in this study as a balanced choice.

### 2.2. Material Properties

This test produces a set of short concrete cylinders made of fiber-reinforced recycled aggregate concrete (FRC50), with 50% of the aggregate replaced by recycled material, and a design strength of 38.1 MPa. The mixing ratios for the FRC50 are shown in Table 2.

The cement used in this study was Bohai brand PO.42.5 ordinary silicate cement. The water-reducing agent was a high-efficiency polycarboxylic acid type with a water-reduction rate exceeding 30%. First-class fly ash produced by Datang Henan Power Generation Co. (Zhengzhou, China) was used as the fly ash material. The BFRP strips had a thickness of 0.110 mm and a width of 300 mm. A two-component epoxy resin impregnation adhesive with a mixing ratio of 2:1 was used. The ultimate tensile strength and modulus of elasticity of the epoxy resin, as provided by the manufacturer, were 55.1 MPa and 2.71 GPa, respectively.

**Figure 1:**
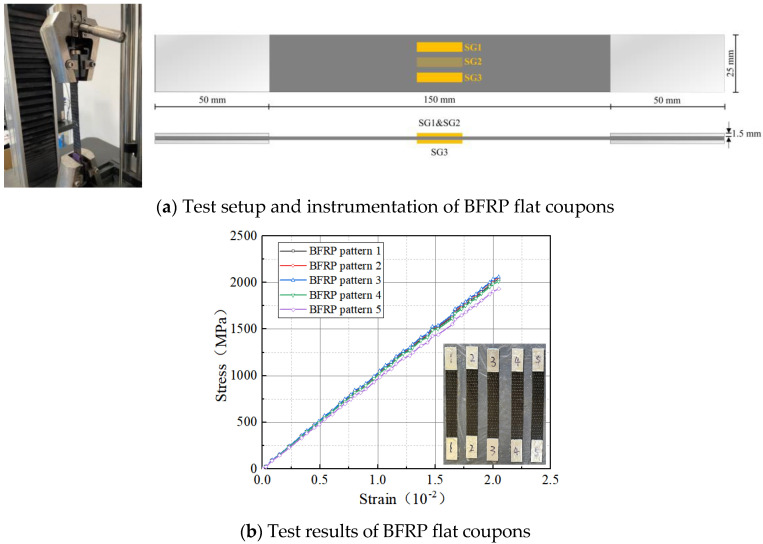
BFRP material.

In this research, the tensile properties of the BFRP specimens were tested in accordance with ASTM D3039M [30]. The test setup and the placement of measurement points are shown in Figure 1a. From the tests, the average modulus of elasticity, ultimate tensile strength, and strain at break of the BFRP cloth were determined to be 87.2 GPa, 1207 MPa, and 0.0251, respectively. The corresponding stress–strain curve is linearly elastic as in Figure 1b. Basalt fibers with a length of 18 mm and a diameter of 13 µm were used in this study. According to the manufacturer’s technical specifications, the fibers exhibit a modulus of elasticity of 91.2 GPa, a tensile strength of 3800 MPa, and an elongation at break of 3.1%.

**Figure 2:**
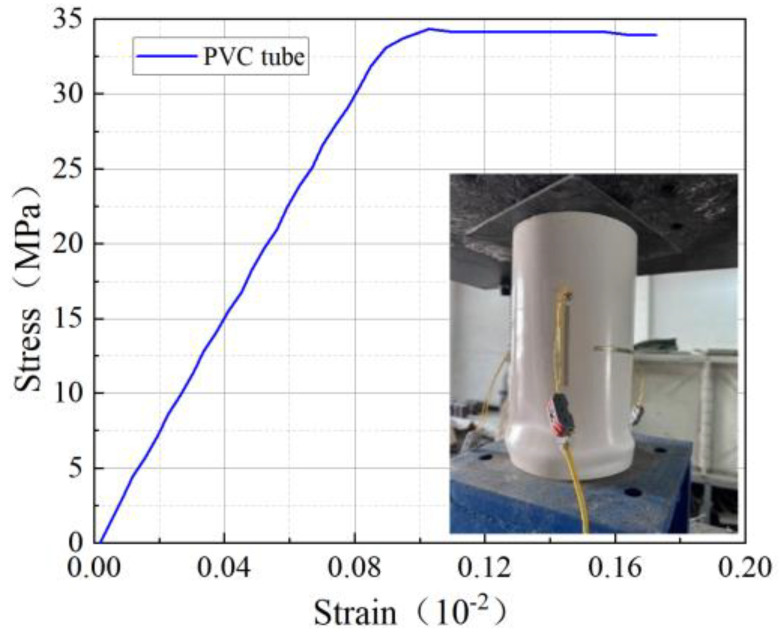
Test results of the PVC tube.

The PVC tubes used in this test were manufactured by Zhejiang Mingde Plastic Technology Co. The performance tests of the PVC tubes were conducted in accordance with the provisions of GB/T 8804.2-2003 [31]. The test results are presented in Table 3, while the tensile stress–strain curves and axial compression damage patterns are shown in Figure 2.

### 2.3. Measurement Procedures

**Figure 3:**
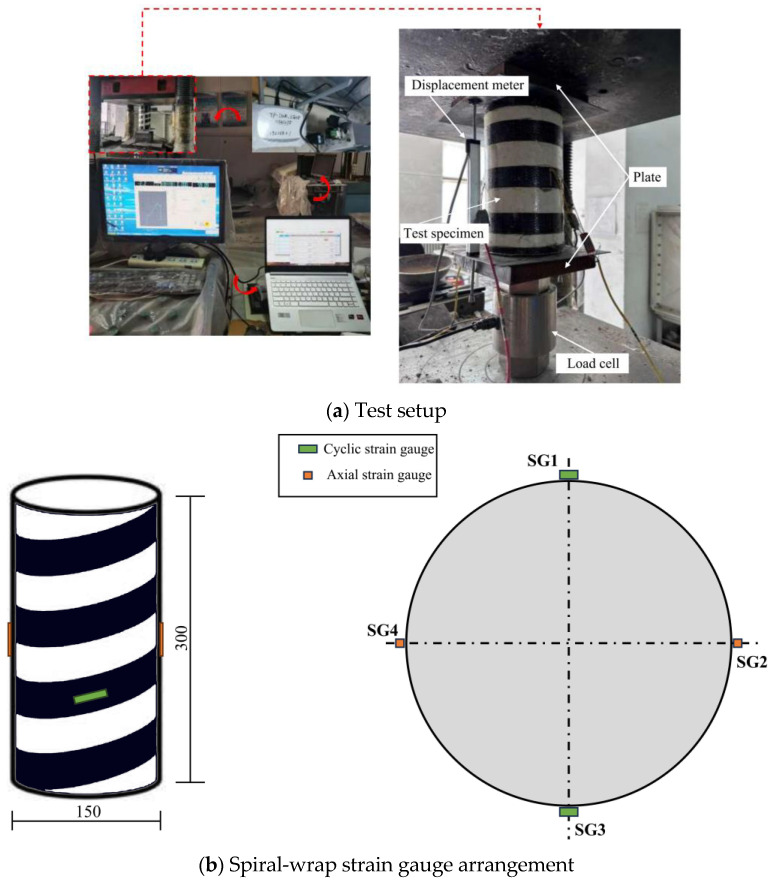
Test procedure.

All experiments in this study were conducted using YAW-5000 microcomputer-controlled voltage servo universal testing equipment (Dawson Group Ltd., Qingdao, China), and the test setup is shown in Figure 3a. The tests were performed under displacement-controlled monotonic loading until failure, with a loading rate of 0.5 mm/min.

The strain measurement in this test was divided into two stages: the attachment of strain gauges (SG) and the placement of displacement gauges (LVDT). For each concrete specimen, four strain gauges (SG1–SG4), each 120 mm in length, were attached at the center of the specimen. Two longitudinal strain gauges, oriented 180° apart, were used to monitor axial strain, while the other two strain gauges, aligned with the helical strip direction, measured circumferential strain, as illustrated in Figure 3b. The load, displacement, and strain data were recorded simultaneously using the dynamic LG08 data acquisition system.

## 3. Experimental Results and Discussion

### 3.1. Failure Model and Experimental Results

**Figure 4:**
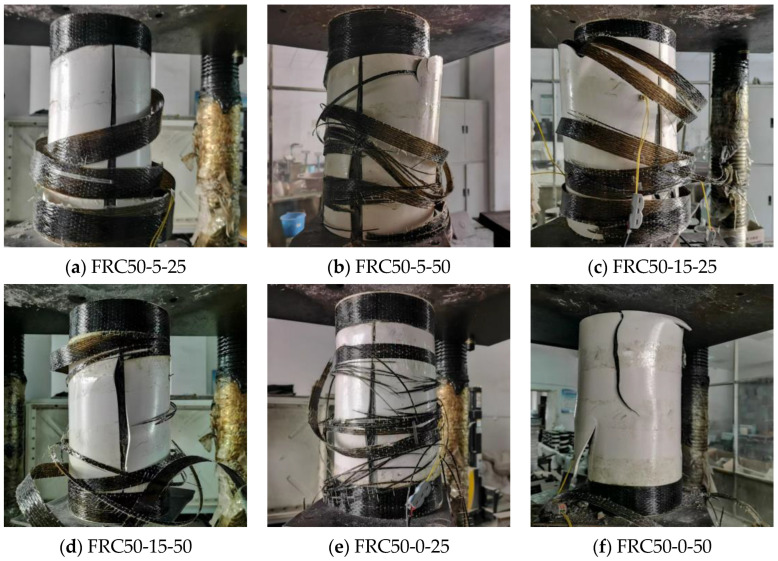
Failure model of BFRP spiral strip–PVC tube confined concrete cylinder.

Through observation, it was found that all confined specimens exhibited similar failure behavior. The BFRP strips fractured at the midsection along the direction of the fiber winding, accompanied by a loud splitting sound from the PVC tubes. Ultimately, the concrete cylinders failed, and the damage patterns are shown in Figure 4.

The damage process can be divided into three stages. In the first stage, at the onset of axial compressive loading, the confined concrete experienced small axial stresses and remained in the elastic stage without significant lateral deformation. In the second stage, as the axial compressive load increased, the core concrete entered the elasto-plastic stage and began to crush internally, leading to pronounced transverse deformation in the central region of the specimen. This deformation caused the PVC tubes at the strip intervals to bulge and curl. In the final stage of loading, the BFRP strips in the central region of the specimen suddenly ruptured completely, and the PVC tubes split, resulting in the loss of lateral confinement on the core concrete. This loss of external constraint caused a sharp drop in the specimen’s load-bearing capacity, bringing the loading process to an abrupt end.

The primary test data are presented in Table 4. The axial stresses, shown on the vertical axes of the graphs, were calculated by dividing the applied load P from the testing machine by the cross-sectional area of the specimen. The axial strain was determined as the average value derived from two LVDT-50 displacement gauges, symmetrically positioned, by dividing the measured displacement by the height of the specimen.

### 3.2. Stress–Strain Curve of the Specimen

**Figure 5:**
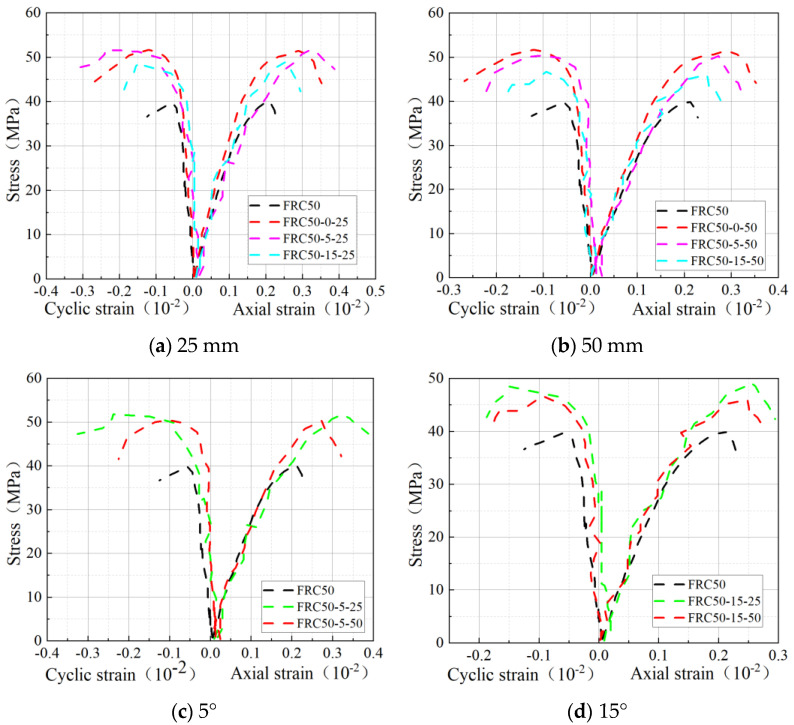
Stress–strain curves of specimens.

As shown in Figure 5a,b, the peak strength and strain of the confined specimens were generally higher than those of the unconfined specimens. For instance, FRC50-5-25 exhibited a modest increase of 0.51% in axial peak strength and a significant increase of 12.21% in axial peak strain compared to FRC50-0-25. This enhancement can be attributed to the lower spiral winding angle being closer to the circumferential winding, which effectively restricts the lateral expansion of concrete, reduces shear deformation, and improves the deformation capacity of the specimens. Conversely, the strength and deformability improvement in specimens with a 15° winding angle was less pronounced compared to those with 5° or circumferential winding. The higher winding angle led to an uneven distribution of the BFRP strips, thereby reducing their restraining effect.

As illustrated in Figure 5c,d, the helical winding pitch significantly influences the strength and deformation capacity of the specimens. A smaller pitch provides a more uniform and effective restraining force, thereby enhancing the concrete’s strength and deformation capacity. For instance, FRC50-5-25 increased the axial peak strength and strain by 29.77% and 52.54%, respectively, compared to the unconfined FRC50 specimens. In comparison, FRC50-5-50 improved these values by 25.96% and 28.47%. Furthermore, FRC50-5-25 demonstrated a 3.03% and 20.61% improvement in strength and strain, respectively, over FRC50-5-50. Similarly, FRC50-15-25 showed a 6.55% increase in strength and a 6.35% increase in strain compared to FRC50-15-50.

### 3.3. Bearing Capacity and Ductility

**Figure 6:**
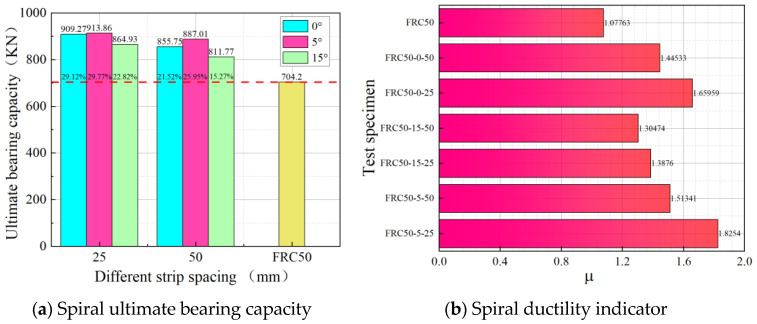
Bearing capacity and ductility.

Figure 6a illustrates the ultimate load-bearing capacity of the different specimens, which decreases with an increase in helix pitch at the same winding angle. Specifically, the load-bearing capacities of FRC50-5-25 and FRC50-5-50 increased by 29.77% and 25.96%, respectively, compared to the unconfined specimens. Similarly, FRC50-15-25 and FRC50-15-50 showed increases of 22.82% and 15.28%, respectively. At the same helix pitch, an increase in the winding angle initially resulted in a slight improvement, followed by a significant reduction in ultimate load-bearing capacity. For instance, FRC50-5-25 demonstrated 1.06 times the load-bearing capacity of FRC50-15-25 and 1.01 times that of FRC50-0-25. It is noteworthy that the load-bearing capacity of the specimens with a winding angle of 5° and small spacing is comparable to that of conventionally annularly restrained concrete.

Figure 6b shows that the ductility of the restrained specimen FRC50-5-25 is significantly better than that of the unrestrained specimen. This indicates that an increase in external confining stress enhances the ductility of the specimen. A reduction in the BFRP helix pitch improves ductility, while an increase in the winding angle reduces it. Therefore, the appropriate design of the winding angle and helix pitch can effectively enhance the plastic deformation capacity of FRP helix-constrained concrete and improve the overall structural ductility. The formula for calculating ductility is given in the following equation:

(1) $$\mu =\frac{\varepsilon_{cu}}{\varepsilon_{co}}$$

### 3.4. BFRP Circumferential Strain Utilization

**Figure 7:**
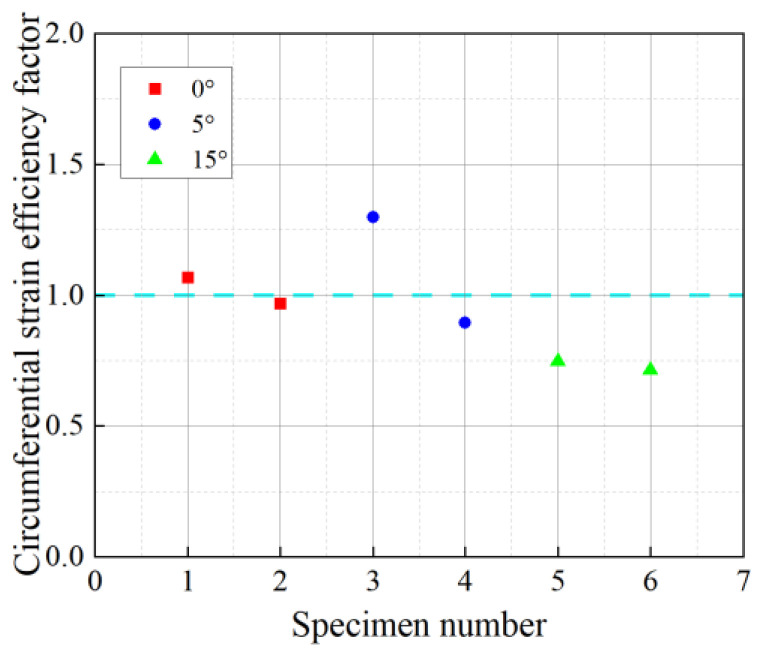
BFRP circumferential strain efficiency factor.

In the confined specimens, although the PVC tube could transmit stress uniformly, the distribution of circumferential strain was uneven due to ineffective restraining zones between the strips. This led to the fracture of the BFRP strips when the local strain approached its limit, while the strain in other regions did not reach the limit, preventing the full utilization of the BFRP material’s capabilities. To address this issue, the FRP circumferential strain efficiency factor $k_{\varepsilon }$ is introduced:

(2) $$k_{\varepsilon }=\frac{\varepsilon_{h,rup}}{\varepsilon_{f}}$$

where $\varepsilon_{f}$ is the ultimate tensile strain determined in the tensile test of BFRP material. For illustrative purposes, the annular strain efficiency coefficients are magnified tenfold in Figure 7. The BFRP strain efficiency coefficients across different confined specimens are observed to be close to 1.0, which is significantly lower than the ultimate tensile strain capacity of BFRP. This suggests that the material’s tensile potential is not fully exploited. The minor variations in the coefficients among specimens indicate that the confinement efficiency of BFRP is largely consistent across all configurations. Accordingly, the actual fracture strain of BFRP should be incorporated into the stress–strain model, and its associated efficiency factor $k_{\varepsilon }$ should be explicitly accounted for.

## 4. FE Analysis

### 4.1. Constitutive Relations of Materials

#### 4.1.1. Concrete

The experimental study was conducted using the Concrete Damage Plasticity (CDP) model in ABAQUS 2024 software, and the CDP model parameters are presented in Table 5. The stress–strain relationship for concrete in the plastic damage model, as defined in GB50010-2010 (2015 edition) [32], is given by the following equation:

(3) $$y=\left\{\begin{array}{cc} \alpha_{a}x+\left(3-2\alpha_{a}\right)x^{2}+\left(\alpha_{a}-2\right)x^{3} & 0\leq x\leq 1\\ \frac{x}{\alpha_{c}{\left(x-1\right)}^{2}+x} & x>1 \end{array}\right.$$

(4) $$x=\frac{\varepsilon }{\varepsilon_{cc}}$$

(5) $$y=\frac{\sigma }{f_{co}}$$

where $\alpha_{a}$ takes the value of 1.65 and $\alpha_{c}$ takes the value of 2.48.

To account for the fiber effect in the simulation, we calibrated the stress–strain curves based on experimental data. First, we obtained the curves for concrete without fibers and then compared them to those of fiber-reinforced concrete (with 0.2% fiber content), focusing on differences in ductility and crack resistance after peak stress. We adjusted the concrete material parameters in the FE model, particularly in tensile strength and strain-hardening behavior, and validated the model by comparing the simulated results with experimental data to ensure accuracy.

#### 4.1.2. PVC Tube

Based on the outcomes of the material characterization tests, the bifold model in ABAQUS was selected because PVC and elastoplastic materials are equivalent [33]. Table 3 provides specific values.

#### 4.1.3. BFRP Strip

As illustrated in Figure 1b, BFRP is considered to be a linear-elastic material, and its cyclic tensile strain reaches the ultimate tensile strain of the fibers before the stress–strain develops linearly. Its specific expression is as follows:

(6) $$\sigma_{f}=\left\{\begin{array}{c} E_{f}\varepsilon_{f}\;0<\varepsilon <\varepsilon_{h,rup}\\ 0\;\varepsilon >\varepsilon_{h,rup} \end{array}\right.$$

where $E_{f}$ is the modulus of elasticity of the BFRP strip, $\varepsilon_{f}$ is the tensile strain of the BFRP strip, $\varepsilon_{h,rup}$ is the ultimate breaking strain of the BFRP strip, and $\sigma_{f}$ is the tensile stress applied to the BFRP strip. The Poisson’s ratio is 0.25.

### 4.2. Grid Division and Cell Selection

**Figure 8:**
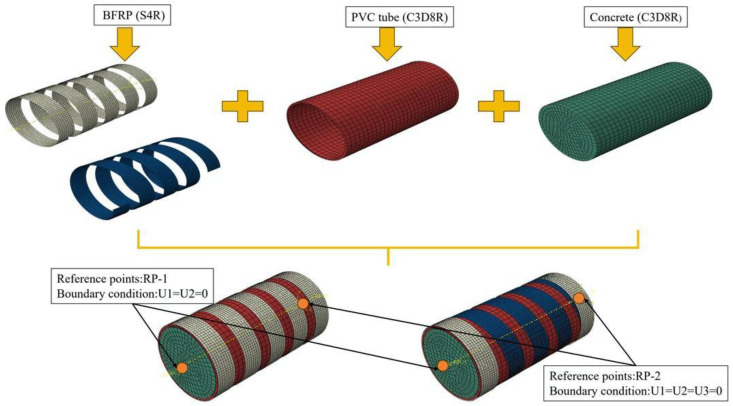
Finite element physical model.

After performing a mesh sensitivity analysis, a 5 mm mesh size was adopted for the BFRP, while a 10 mm mesh size was used for the PVC and core concrete. The finite element model is shown in Figure 8. The core concrete and PVC tube are modeled using C3D8R elements, while the BFRP is modeled using S4R elements. For the shell element, Simpson’s integration with three integration points in the thickness direction was selected.

### 4.3. Boundary Conditions and Interactions

The boundary conditions were defined by restraining the displacements of two reference points, RP1 and RP2, located on the top and bottom loading plates, respectively. RP2 was fully fixed in all degrees of freedom, while RP1 was constrained in rotation and lateral displacement, allowing movement only in the *Z*-axis direction to apply axial load. To simulate the interactions between different components—namely, the concrete core, PVC tube, and spiral BFRP strips—surface-to-surface contact was employed. In all cases, the contact in the normal direction was defined using a hard contact formulation to prevent interpenetration, while the tangential behavior was modeled using a penalty friction formulation. A friction coefficient of 0.25 [28,33] was assigned for both the concrete–PVC and concrete–BFRP interfaces. This value corresponds to a partially bonded interface, which allows limited sliding between surfaces while maintaining sufficient resistance to relative displacement under loading. The selection of this value is consistent with previous studies [33,34] involving similar material combinations and provides a balance between numerical stability and realistic confinement interaction. No cohesive or perfect bond models were used in this simulation, as the main focus was on the mechanical confinement effects provided by the external BFRP and PVC, rather than on detailed bond-slip behavior.

### 4.4. FE Models Validation

**Figure 9:**
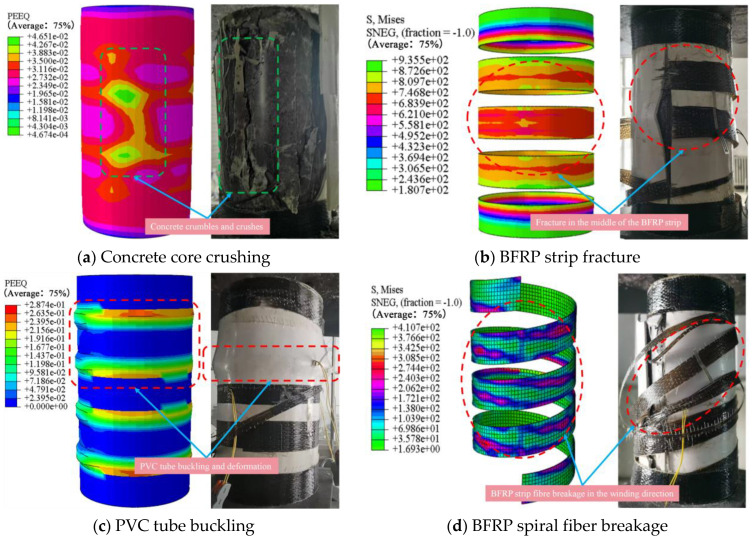
Comparison of failure models.

In this study, finite element simulations were conducted for specimens FRC50-0-25, FRC50-0-50, FRC50-5-25, and FRC50-5-50, and the results were validated against experimental data. Representative comparative graphs were selected for analysis to evaluate the accuracy and performance of the numerical models. Figure 9a illustrates the failure of the concrete core. The finite element model shows a central hourglass-shaped zone of high plastic strain, indicating shear-dominated damage typical of axially compressed cylinders. Experimental results confirm severe spalling and crushing in the same region, suggesting that axial stress concentration and the heterogeneity of recycled concrete lead to localized failure governing the overall compressive behavior. Figure 9b presents the stress distribution in the BFRP spiral. The simulation identifies a peak stress concentration at the mid-span of the spiral loop, which aligns with experimental evidence of fiber breakage at this location. This indicates that bending and tensile forces from lateral dilation critically affect spiral performance, emphasizing the role of geometry and spacing in spiral failure. Figure 9c shows the deformation of the PVC tube under compression. Periodic strain bands predicted by the simulation correspond to buckling zones between spiral turns. Experimental observations reveal similar wrinkling, indicating that while PVC offers limited confinement, it interacts with the spiral through localized deformation, helping delay concrete expansion. Figure 9d displays von Mises stress distribution along the BFRP spiral. High tensile stress aligned with the fiber direction leads to rupture once the tensile strength is exceeded. This failure mode highlights the importance of fiber orientation and stress paths in determining the structural integrity of the spiral. Overall, the finite element simulations effectively capture the principal deformation and failure mechanisms observed in the experiments. The PVC tube experienced localized buckling under radial pressure, the recycled concrete core failed through shear-dominated crushing near mid-height, and the BFRP spirals exhibited both mid-span breakage due to bending–tension interaction and fiber rupture along the winding direction. These results confirm that the mechanical response of the composite confined concrete system is governed by the complex interaction among the concrete core, spiral reinforcement, and the PVC tube. The simulations not only validate the experimental findings but also offer in-depth insights into the evolution of stress and strain fields that lead to structural failure, thereby providing a valuable foundation for the design and optimization of composite confinement systems.

**Figure 10:**
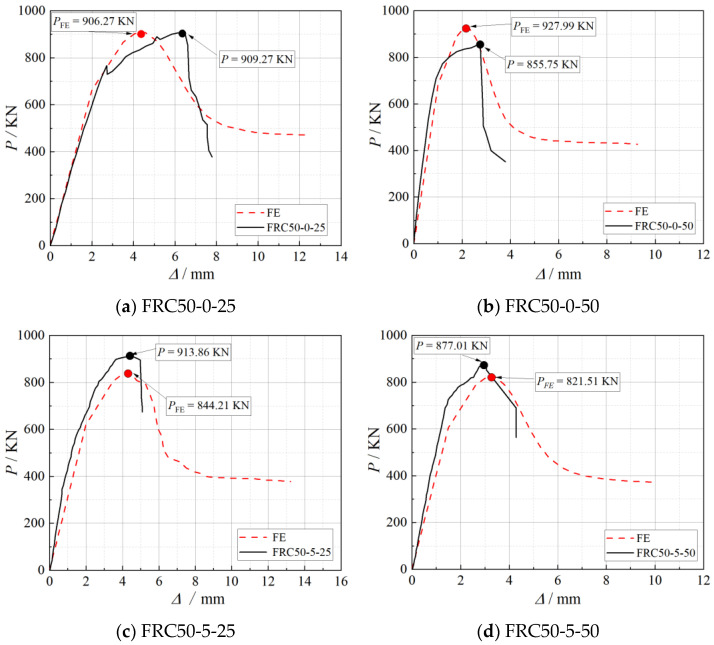
Load–displacement curve comparison.

Figure 10 and Table 6 demonstrate that both the finite element (FE) analysis and the experimental tests yield nonlinear load–displacement responses. In the initial loading stage, the FE results closely follow the experimental trends, and the predicted peak loads ($P_{FE}$) are in good agreement with the measured values (P). However, after the onset of damage, significant discrepancies emerge between the two. The FE curves fail to adequately capture the post-peak softening and ductility exhibited in the experimental tests.

This discrepancy can be attributed to several factors. First, the concrete was modeled using the Concrete Damaged Plasticity (CDP) model, which, while suitable for capturing general nonlinear behavior, may not fully replicate the complex damage evolution, cracking, and energy dissipation occurring in actual specimens. Second, the short basalt fibers mixed into the concrete matrix were not explicitly modeled; their reinforcing effect was incorporated implicitly by calibrating the concrete’s stress–strain curve, which may underestimate their contribution to ductility, especially in the post-peak regime. Third, the contact interfaces between concrete, PVC, and BFRP strips were simplified as frictional surfaces with fixed coefficients, without modeling progressive debonding or bond-slip behavior that may occur during failure. Lastly, experimental variability and microstructural inconsistencies—such as uneven fiber dispersion, initial microcracks, or local failure modes—may also contribute to the observed differences.

Despite these limitations, the FE model effectively captures the overall load–displacement trend and peak load behavior, demonstrating its usefulness for predicting the general performance of the composite system under axial compression.

## 5. Strength Prediction Model

### 5.1. Calculation of Effective Lateral Restraint Stress

Because the PVC tube is subject to biaxial stress, it will experience both axial and circumferential stresses ($f_{lp}$). The circumferential confining stress of the PVC tube can be calculated using the following formula:

(7) $$f_{lp}=\frac{2E_{P}\varepsilon_{P}t_{P}}{D+2t_{p}}$$

where $t_{P}$ is the thickness of the PVC tube; $E_{P}$ is the modulus of elasticity of the PVC tube; and $\varepsilon_{P}$ is the ultimate tensile strain of the PVC tube; D is the diameter of the short cylinder.

In a fully encased confined concrete cylinder, the lateral restraining stress ($f_{lf}$) provided by FRP can be assumed to be uniformly distributed around the circle. The restraining effect of FRP on core concrete is passive [34]. According to the force equilibrium relationship, A can be calculated as a function of the ultimate tensile strain ($\varepsilon_{f}$) by using Equation (8).

(8) $$f_{lf}=\frac{{2E}_{f}\varepsilon_{f}t_{f}}{D}$$

where $t_{f}$ is the thickness of the BFRP strip. To increase the accuracy of the design model, Lam and Teng [35] suggested using the actual FRP annular fracture strain $\varepsilon_{h,rup}$ to determine the actual lateral confining pressure of FRP. The incorporation of the FRP circumferential strain efficiency factor $k_{\varepsilon }$ in Equation (1) to indicate the negative influence on the restraint effect fits with the hypothesis of Lam and Teng. It is calculated as follows:

(9) $$\varepsilon_{h,rup}=k_{\varepsilon }\varepsilon_{f}$$

**Figure 11:**
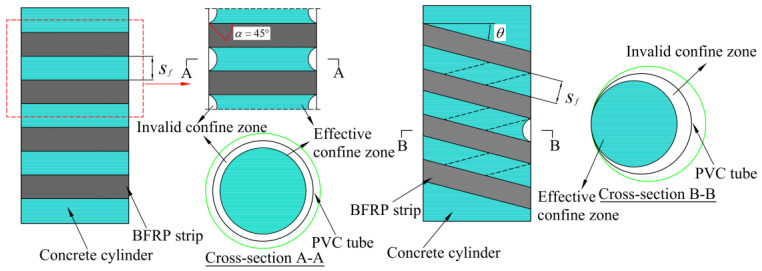
Mechanism of concrete cylinder confined by BFRP strip–PVC tube.

The Italian specification employs the case of the “arch effect” [36], a hypothesis that uses a parabola whose initial tangent is at 45° to the horizontal to differentiate between areas of effective constraints and areas of ineffective constraints, as shown in Figure 11. By considering the influence of partial restraint of FRP strips, the vertical restraint coefficient $k_{v}$ associated with the strip restraint is proposed to reflect the relationship between the effective restrained concrete area and the cross-sectional area of the concrete cylinder, and the equations for the circumferential restraint and spiral restraint are as follows:

(10) $$k_{v}=\left\{\begin{array}{cc} {\left(1-\frac{s_{f}}{2D}\right)}^{2} & circumferential\\ 1-\frac{s_{f}}{2D} & spiral \end{array}\right.$$

where $s_{f}$ is the strip spacing or helix pitch. For spiral strips confining concrete cylinders, additional restraint effect coefficient $k_{\theta }$, related to the winding angle θ, should also be addressed.

(11) $$k_{\theta }=\frac{1}{1+{tan}^{2}\theta }$$

Therefore, the actual lateral confining stress $f_{l}^{\prime }$ provided by the BFRP strip is calculated as follows:

(12) $$f_{lf}^{\prime }=\frac{{2k}_{v}k_{\theta }k_{\varepsilon }E_{f}\varepsilon_{f}t_{f}}{D}$$

That is, the lateral confinement force ($f_{l}$) provided by the external confine is computed as follows:

(13) $$f_{l}=f_{lf}^{\prime }+f_{lp}=\frac{{2k}_{v}k_{\theta }k_{\varepsilon }E_{f}\varepsilon_{f}t_{f}}{D}+\frac{2E_{P}\varepsilon_{P}t_{P}}{D+2t_{p}}$$

### 5.2. Strength Modeling of Typically Constrained Concrete Cylinders

**Figure 12:**
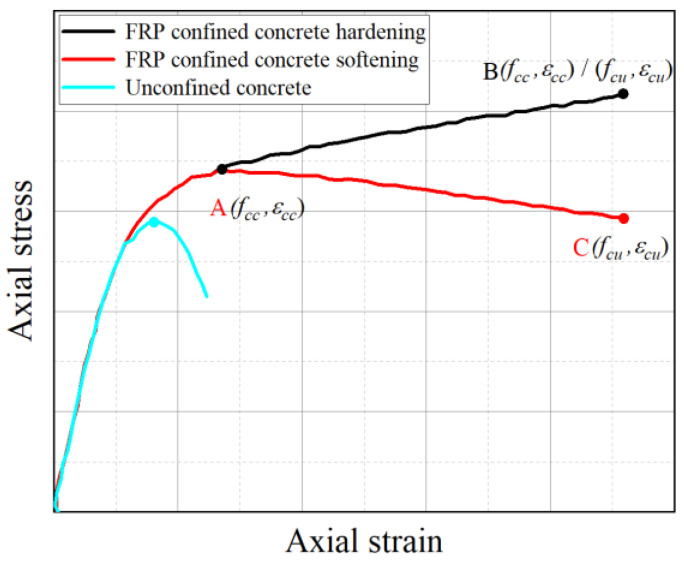
Typical axial stress–strain curve of FRP-confined concrete.

Figure 12 illustrates the typical axial stress–strain behavior of FRP-confined concrete. The model assumes that the initial ascending branch follows a trend similar to that of unconfined concrete, with the enhancement effect of the FRP becoming apparent near the inflection point A. Upon entering the transition zone, the rapid lateral expansion of the concrete activates the passive confinement provided by the FRP, generating lateral stresses that mitigate axial stiffness degradation and help maintain the integrity of the concrete core. This confinement leads to the development of a secondary branch in the curve. When the FRP confinement level exceeds a certain threshold, the post-peak response exhibits strain hardening (line A–B); otherwise, strain softening occurs (line A–C).

The axial compression tests conducted in this study reveal that the stress–strain curves of BFRP strip–PVC tube confined concrete cylinders display characteristics of relatively weak confinement, irrespective of whether circumferential or spiral wrapping is applied. This weak confinement effect is primarily attributed to factors such as larger strip spacing, relatively low winding angles, limited thickness and stiffness of the PVC tubes and BFRP strips, as well as possible imperfect bonding at the interfaces.

To address these limitations, this study proposes a modified stress–strain model developed through localized refinement of existing design-oriented models. The modification synthesizes previous models and incorporates key parameters such as strip spacing and winding angle, identified through representative stress–strain behavior analyses. Additionally, engineering measures—such as increasing winding angle, reducing strip spacing, enhancing material stiffness, and improving bonding quality—are recommended to improve confinement efficiency and enhance the mechanical performance of these composite concrete cylinders.

### 5.3. Evaluation and Analysis of Existing Models

Jiang et al. [37], for weakly constrained specimens, proposed to consider the effects of two parameters, the constrained stiffness ratio ($\rho_{k}$) and the strain rate ($\rho_{\varepsilon }$), in the strength model and the ultimate axial strain model, respectively, and corrected the bounding values for judging the strong/weak constraints by regressing the test data:

(14) $$\frac{f_{cu}}{f_{co}}=\left\{\begin{array}{c} 1+3.5(\rho_{k}-0.01)\rho_{\varepsilon },\;if\;\rho_{k}\geq 0.01\\ 1,\;if\;\rho_{k}<0.01 \end{array}\right.$$

(15) $$\frac{\varepsilon_{cu}}{\varepsilon_{co}}=1.65+6.5\rho_{k}^{0.8}\rho_{\varepsilon }^{1.45}$$

(16) $$\rho_{k}=\frac{2E_{f}\varepsilon_{f}}{(f_{co}/\varepsilon_{co})D},\rho_{\varepsilon }=\frac{\varepsilon_{h,rup}}{\varepsilon_{co}}$$

Based on the model of Jiang et al. [37] and considering the “arch effect” theory as well as the influence of strip spacing on the FRP-constrained stiffness ratio, Guo et al. [38] proposed using the constraint stiffness ratio ($\rho_{k}$) of fully wrapped restrained concrete, multiplied by the vertical effectiveness factor ($k_{v}$), to obtain the effective constraint stiffness ratio ($\rho_{ke}$) for a horizontally annular, partially confined concrete cylinder with FRP strips. The equation used for calculation is as follows:

(17) $$\rho_{ke}=k_{v}\rho_{k}=k_{v}\frac{2E_{f}t_{f}}{\left(f_{co}/\varepsilon_{co}\right)D}$$

**Figure 13:**
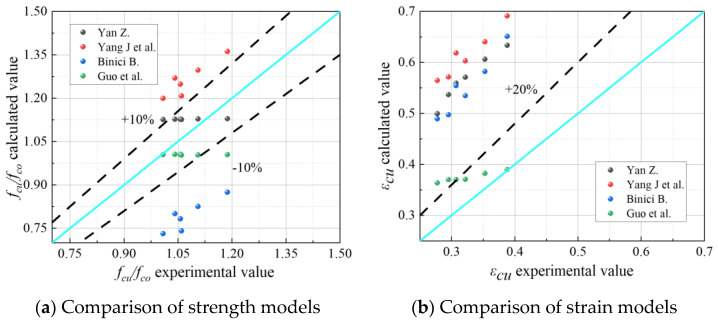
Model comparison [38,39,40,41].

However, due to the different number of specimens, different forms of constraints in the databases used by different researchers, and a certain degree of dispersion in the test results, the coefficients in the expressions of each model are different, as shown in Table 7, and the results of the analyses are shown in Figure 13.

Based on the experimental data from this study, the degree of deviation between the design and experimental values of strength and ultimate axial strain for each model is evaluated. Three performance indicators were used for the evaluation: root mean square error (RMSE), coefficient of variation (CoV), and average absolute error (AAE). The equations used for calculation are presented below:

(18) $$RMSE=\sqrt{\frac{\sum_{i=1}^{n}{\left(y_{i}-y_{oi}\right)}^{2}}{n}}$$

(19) $$CoV=\frac{\sqrt{\frac{\sum_{i=1}^{n}{\left(\frac{y_{i}}{y_{oi}}-MEAN\right)}^{2}}{n}}}{MEAN}$$

(20) $$AAE=\frac{\sum_{i=1}^{n}\left|\frac{y_{i}-y_{oi}}{y_{oi}}\right|}{n}$$

(21) $$MEAN=\frac{\sum_{i=1}^{n}\frac{y_{i}}{y_{oi}}}{n}$$

where n signifies the number of data; i denotes group i input data; $y_{i}$ denotes the predicted value; $y_{oi}$ denotes the experimental value; MEAN denotes the mean value.

The results of the assessment indicators of the design model are shown in Table 8. From the above comparative studies, it can be shown that (1) due to the consideration of the FRP strip annular confinement efficiency coefficients, the strength model prediction results of Yan Z. [38] are in good agreement; (2) due to the consideration of the effects of strip spacing on the FRP confinement effect, the effective confinement stiffness value is employed, and the anticipated findings of the ultimate strain model of Guo et al. [37] are in better agreement compared to the other models; (3) existing models still have a considerable bias in forecasting the strength of restrained concrete in the case of weak restraint.

### 5.4. Model Modification and Validation

This study focuses on the weak confinement effect of BFRP spiral strip–PVC tube confined concrete cylinders, particularly in the presence of softening segments in the stress–strain curve. Therefore, the strength model proposed by Yan Z. [39] is used to determine the validity of the external confinement based on $f_{l}^{\prime }/f_{co}$. The ultimate axial strain, on the other hand, is related to two parameters: the constrained stiffness ratio ($\rho_{k}$) and the strain rate ($\rho_{\varepsilon }$), which are selected to correct $\rho_{k}$ according to the model of Guo et al. [38], as follows:

(22) $$\rho_{ke}=k_{v}k_{\theta }\rho_{k}=(1-\frac{s_{f}^{\prime }}{2D})\frac{E_{f}t_{f}}{2(1+{tan}^{2}\theta )(f_{co}/\varepsilon_{co})}$$

(23) $$\frac{f_{cu}}{f_{co}}=a\left(\frac{f_{l}^{\prime }}{f_{co}}\right)+b$$

(24) $$\varepsilon_{cu}=c+d{{(\rho }_{ke}\frac{s_{f}+b_{f}}{b_{f}})}^{e}\rho_{\varepsilon }^{f}$$

Therefore, the above expression was analyzed by regression from the experimental data, and the coefficients a, b, c, d, e, and f were modified as shown in the following equation:

(25) $$\frac{f_{cu}}{f_{co}}=1.13\left(\frac{f_{l}^{\prime }}{f_{co}}\right)+1.01$$

(26) $$\varepsilon_{cu}=0.27+3.5{{(\rho }_{ke}\frac{s_{f}+b_{f}}{b_{f}})}^{0.82}\rho_{\varepsilon }^{1.53}$$

**Figure 14:**
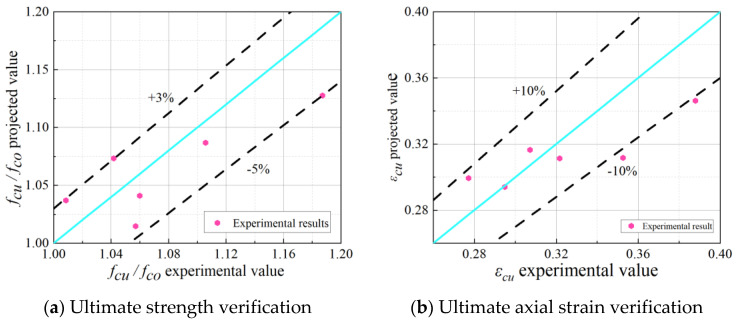
Comparison of model results.

Figure 14 compares the predicted and tested values of the proposed strength model and the ultimate axial strain model. The error of the strength model ranges from +3% to −5%, while the error of the ultimate axial strain model is within ±10%. These errors show good agreement with the test data.

## 6. Conclusions

A new winding method for BFRP-PVC confined RAC cylinders was developed to provide a novel structure for future practical applications. The structure underwent axial compression tests and ABAQUS finite element analysis to evaluate its axial compression performance. The following conclusions were drawn:

(1) Under the composite system of the new winding method, a reasonable winding angle and strip spacing effectively enhanced the bearing capacity. Compared to the unconfined specimen, the peak axial bearing capacity of the confined specimen was increased by 15.28% to 29.77%.
(2) The one-piece nature of the spiral wound strip significantly improved the stress–strain curve of the specimen. At a reasonable winding angle, the axial and circumferential strains of the best spirally wound specimen increased by 12.21% and 21.62%, respectively, compared to the best circumferentially wound specimen.
(3) A well-designed winding angle and helix pitch can improve the plastic deformation capacity of the reinforced specimens and enhance structural ductility. The ductility index of the best spirally wound specimen was 1.83, which is 69.39% higher than the unconfined specimen and 10% higher than the best circumferentially wound specimen.
(4) The finite element model of BFRP spiral strip–PVC tube confined RAC was established using ABAQUS finite element analysis software. The simulated damage morphology closely matches the actual observations, validating the modeling approach.
(5) A stress–strain model was developed by correcting existing models and fitting the experimental data. The error of the strength model ranged from +3% to −5%, and the error of the ultimate axial strain model was within ±10%.

This study acknowledges a limitation in that the effect of basalt fibers was not explicitly modeled in the finite element simulations. Instead, the fibers’ contribution was indirectly included by calibrating the concrete’s stress–strain parameters to account for improvements in tensile resistance and ductility due to fiber reinforcement. While this approach made the model more computationally efficient, it may limit the accuracy of the results, particularly in capturing the exact role of the fibers in the behavior of confined concrete. This limitation should be considered when interpreting the results, especially regarding fiber–matrix interaction. Future studies could improve accuracy by incorporating explicit fiber modeling in finite element simulations.

## Figures and Tables

**Table 1:** Specific parameters of the spiral wrap specimen.

Test Specimen	Recycled Aggregate Substitution Rate (%)	Helix Pitch (mm)	Strip Layer	PVC Tube	Number of Individuals	Winding Angle (◦)
FRC50-5-25	50	25	1	1	3	5
FRC50-5-50	50	50	1	1	3	5
FRC50-15-25	50	25	1	1	3	15
FRC50-15-50	50	50	1	1	3	15
FRC50-0-25	50	25	1	1	3	0
FRC50-0-50	50	50	1	1	3	0
FRC50	50	/	/	/	3	/

**Table 2:** Proportion of concrete.

Classify	Clinker (kg/m^3^)	Granule (kg/m^3^)	Natural Aggregate (kg/m^3^)	Recycled Aggregate (kg/m^3^)	Coal Ash (kg/m^3^)	Water-Reducing Agent (kg/m^3^)	Water (kg/m^3^)	Fiber Content (%)
FRC50	414	626	556	556	138	1.81	160	0.2

**Table 3:** Properties of PVC tube.

Model Number	Density (kg/m^3^)	Tensile Strength (MPa)	Modulus of Elasticity (GPa)	Elongation at Break (10^−2^)	Poisson’s Ratio	Compressive Strength (MPa)
D160 × 4	1350–1550	34.11	3.32	1.03	0.38	48.3

**Table 4:** BFRP spiral strip–PVC tube confined specimen results.

Test Specimen	P (KN)	$f_{cc}$ (MPa)	$f_{cu}$ (MPa)	$f_{co}$ (MPa)	$\varepsilon_{co}$ (10^−2^)	$\varepsilon_{cc}$ (10^−2^)	$\varepsilon_{cu}$ (10^−2^)	$\varepsilon_{h,rup}$ (10^−2^)
FRC50-5-25	913.86	51.74	47.33	39.87	0.21254	0.32420	0.38797	0.32589
FRC50-5-50	887.01	50.22	42.15	39.87	0.21254	0.27306	0.32166	0.22466
FRC50-15-25	864.93	48.97	42.26	39.87	0.21254	0.25568	0.29492	0.18729
FRC50-15-50	811.77	45.96	40.21	39.87	0.21254	0.24845	0.27731	0.17935
FRC50-0-25	909.27	51.48	44.09	39.87	0.21254	0.28891	0.35273	0.26796
FRC50-0-50	855.75	48.45	41.54	39.87	0.21254	0.27818	0.30719	0.24282
FRC50	704.20	39.87	36.27	39.87	0.21254	0.21254	0.22904	0.12506

Note: P is the ultimate bearing capacity of the specimen; $f_{cc}$ is the peak axial stress; $f_{cu}$ is the axial ultimate stress; $f_{co}$ is the peak axial stress of the unconfined specimen; $\varepsilon_{co}$ is the peak axial strain of the unconstrained specimen; $\varepsilon_{cc}$ is the peak axial strain; $\varepsilon_{cu}$ is the axial ultimate strain; and $\varepsilon_{h,rup}$ is the circumferential ultimate strain.

**Table 5:** CDP model parameters.

Swell Angle	Eccentricity	Biaxial to Uniaxial Compressive Strength Ratio	Yield Surface Parameters	Coefficient of Viscosity
30	0.1	1.16	0.667	10^−5^

**Table 6:** Peak load discrepancy analysis for various specimens.

Specimens	Experimental Peak Load (KN)	FE Simulation Peak Load (KN)	Discrepancy (%)
FRC50-0-25	906.27	909.27	0.33
FRC50-0-50	927.99	855.75	7.79
FRC50-5-25	913.86	844.21	7.63
FRC50-5-50	877.01	821.51	6.33

**Table 7:** Existing design models.

Documentation	$f_{cu}$	$\varepsilon_{cu}$
Guo et al. [38]	$\frac{f_{cu}}{f_{co}}=1+2{(\rho }_{k}\frac{s_{f}+b_{f}}{b_{f}}-0.01)\rho_{\varepsilon }$	$\frac{\varepsilon_{cu}}{\varepsilon_{co}}=1.75+5.5{\left(\rho_{k}\frac{s_{f}+b_{f}}{b_{f}}\right)}^{0.8}\rho_{\varepsilon }^{1.45}$
Yan Z. [39]	$\frac{f_{cu}}{f_{co}}=4.721\sqrt{4.193\frac{f_{l}}{f_{co}}+1}-2\frac{f_{l}}{f_{co}}-4.322$ if $\frac{f_{l}}{f_{co}}\geq 0.2$ $\frac{f_{cu}}{f_{co}}=0.0768(\frac{f_{l}}{f_{co}})+1.122$ if $\frac{f_{l}}{f_{co}}<0.2$	$\varepsilon_{cu}=\frac{f_{cu}(1+2\beta_{1}\varepsilon_{h,rup})}{E_{c1}}$ $\beta_{1}=190{\left(\frac{f_{l}}{f_{co}}\right)}^{-0.8}$ $E_{c1}=5500\sqrt{f_{co}}$
Binici B. [40]	$\frac{f_{cu}}{f_{co}}=1+2.6{\left((\frac{f_{l}}{f_{co}})-0.14\right)}^{0.17}$ if $\frac{f_{l}}{f_{co}}\geq 0.14$ $\frac{f_{cu}}{f_{co}}=1.8{\left(\frac{f_{l}}{f_{co}}\right)}^{0.3}$ if $\frac{f_{l}}{f_{co}}<0.14$	$\frac{\varepsilon_{cu}}{\varepsilon_{co}}=1.75+12(\frac{f_{l}}{f_{co}}){\left(\frac{\varepsilon_{h,rup}}{\varepsilon_{co}}\right)}^{0.45}$
Yang J et al. [41]	$\frac{f_{cu}}{f_{co}}=1+4.0\frac{f_{l}}{f_{co}}$	$\frac{\varepsilon_{cu}}{\varepsilon_{co}}=1.75+3\rho_{k}^{0.66}\rho_{\varepsilon }^{1.90}$ (strong constraint) $\frac{\varepsilon_{cu}}{\varepsilon_{co}}=2+24\rho_{k}^{0.68}\rho_{\varepsilon }^{1.08}$ (weak constraint)

Note: $E_{l}$ is the lateral confinement stiffness; $E_{c1}$ is the concrete modulus of elasticity; and $b_{f}$ is the strip width.

**Table 8:** Assessment of the results of the indicators.

Document	RMSE		AAE		CoV	
	$\frac{f_{cc}}{f_{co}}$	$\varepsilon_{cu}$	$\frac{f_{cc}}{f_{co}}$	$\varepsilon_{cu}$	$\frac{f_{cc}}{f_{co}}$	$\varepsilon_{cu}$
Yan Z. [39]	0.075	0.244	0.066	0.761	0.050	0.038
Yang J et al. [41]	0.188	0.291	0.174	0.909	0.022	0.050
Binici B. [40]	0.285	0.229	0.264	0.707	0.029	0.033
Guo et al. [38]	0.092	0.070	0.065	0.204	0.051	0.103

## Data Availability

The original contributions presented in this study are included in the article. Further inquiries can be directed to the corresponding author.
